# *Rhipicephalus sanguineus* sensu lato infestation in an urban area in South Sacramento, California, USA

**DOI:** 10.1186/s13071-025-07069-3

**Published:** 2025-10-27

**Authors:** Mario Novelo, Whitney Clack, Megan Siefker, James Brodigan, Francesca Rubino, Janet Foley, Sarah S. Wheeler

**Affiliations:** 1 Sacramento–Yolo Mosquito and Vector Control District, Elk Grove, CA 95624 USA; 2 School of Veterinary Medicine, Department of Medicine and Epidemiology, University of California, Davis, CA 95616 USA

**Keywords:** Brown dog ticks, *Rhipicephalus sanguineus* sensu lato, *Rickettsia rickettsii*, Rocky Mountain spotted fever, Tick control

## Abstract

**Background:**

The brown dog tick, *Rhipicephalus sanguineus* sensu lato (*Rh. sanguineus* s.l.), is an important vector of *Rickettsia rickettsii*, the causative agent of Rocky Mountain spotted fever (RMSF), in western North America, with the most prominent tick infestations occurring in the Southwestern USA and Northern Mexico. RMSF is a significant public health threat in these regions, including in the state of California.

**Methods:**

In 2024, the Sacramento-Yolo Mosquito and Vector Control District detected a brown dog tick infestation in a neighborhood in South Sacramento (CA, USA) that encompassed three adjoining properties. This infestation was unusual due to its location farther north than those in most recent reports. In partnership with the Laboratory of Infectious Disease Ecology at the University of California, Davis, a surveillance and abatement program was implemented. This included tick monitoring, residual spraying of acaricide, deployment of tick collars on dogs and bilingual public outreach.

**Results:**

The integrated intervention substantially reduced tick populations at the affected site. Both adult and immature stages of *Rh. sanguineus* s.l. declined following sequential treatments. Sustained suppression and elimination were achieved through continued control and outreach efforts.

**Conclusions:**

This localized infestation of* Rh. sanguineus* s.l. in northern California highlights the potential for range expansion of RMSF vectors and underscores the need for continued surveillance, rapid response and community engagement to mitigate vector-borne disease risks.

**Graphical Abstract:**

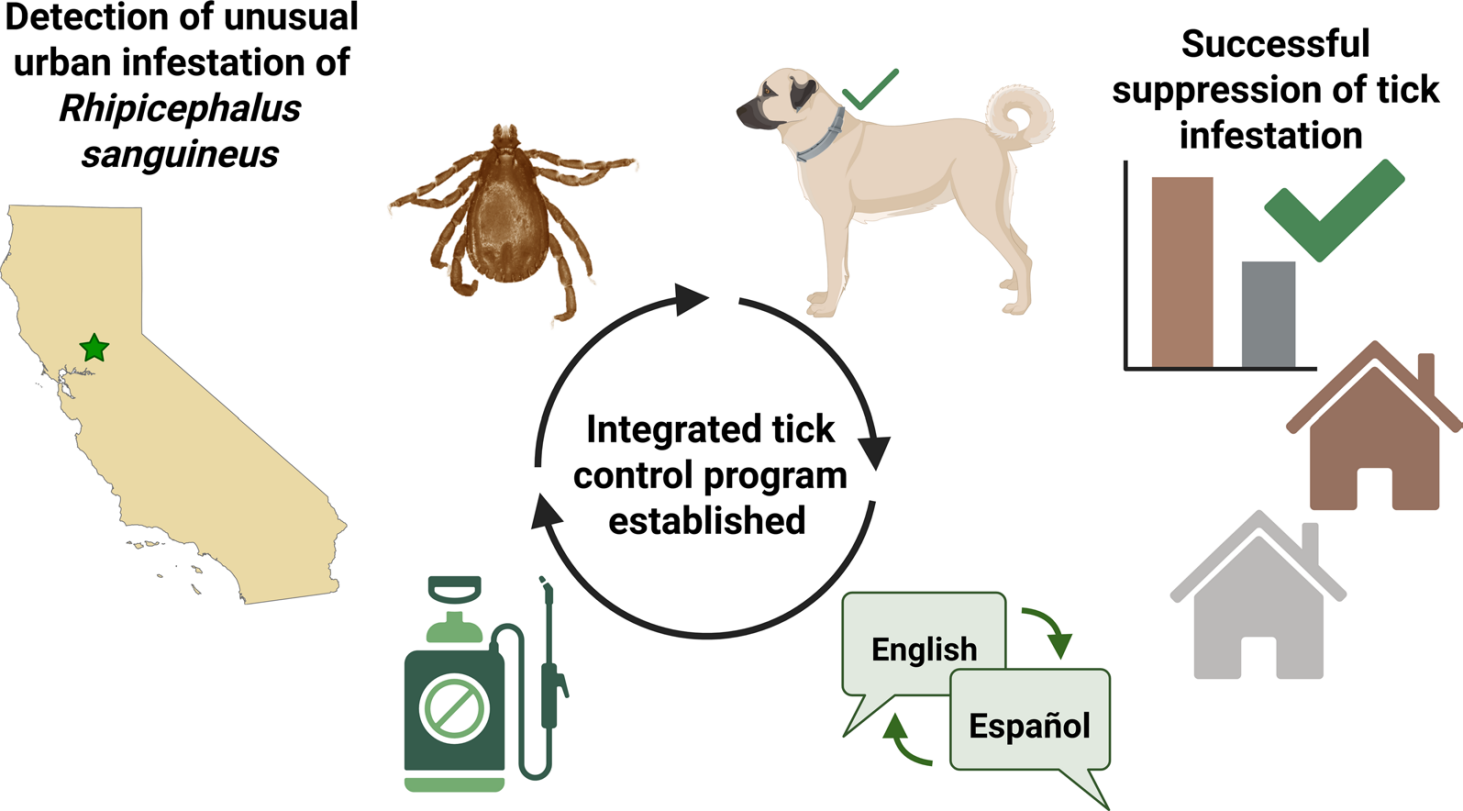

**Supplementary Information:**

The online version contains supplementary material available at 10.1186/s13071-025-07069-3.

## Background

The brown dog tick (BDT; *Rhipicephalus sanguineus* sensu lato) (*Rh. sanguineus* s.l.) is one of the most widespread tick species globally [1] and a known vector of several medically important pathogens, including *Rickettsia rickettsii*, the causative agent of Rocky Mountain spotted fever (RMSF) [2, 3]. This tick thrives both indoors and outdoors, primarily feeding on dogs but occasionally parasitizing other animals, including humans [4–7]. In areas with acute RMSF transmission, infected dogs may act as amplifying hosts, influencing bacterial prevalence in local tick populations [8–11]. While RMSF is relatively rare in California and typically associated with travel to hyperendemic areas like northern Mexico, reports from the California Department of Public Health (CDPH) suggest an increase in human cases, especially among Hispanic residents [12–14]. Other important potential vectors for RMSF in northern California are ticks of the *Dermacentor* genus, including *Dermacentor similis* and *Dermacentor occidentalis* [13].

The BDT will typically display a host–seeking behavior that involves actively hunting by crawling around walls, cracks and crevices of dog and human dwellings, searching for volatile chemicals produced by potential hosts [15]. Under optimal conditions, the species can complete up to four generations annually, exacerbating localized infestations and increasing exposure risk for both dogs and humans to diseases such as RMSF [16]. RMSF remains the most common rickettsiosis in the Southwestern USA [17, 18] and poses serious health risks to both humans and dogs. Caused by the obligate intracellular bacterium *R. rickettsii*, RMSF is often difficult to diagnose and, if left untreated, can rapidly become life-threatening, with pre-antibiotic mortality rates reaching up to 80% and in some areas, such as northern Mexico, having current case-fatality rates of up to 50% [11, 14, 19]. Symptoms typically begin 3–12 days after tick exposure and include fever, myalgia and rash. No vaccines are currently available for humans or dogs [9, 20, 21]; however, early treatment with tetracycline antibiotics, ideally within the first 5 days, can be highly effective in limiting disease severity [20, 22].

Given the complexity of the *R. rickettsii* transmission cycle, which involves humans and dogs and is influenced by environmental factors, a One Health approach is essential to prevent pathogen transmission and BDT infestations [23, 24]. Successful intervention requires collaboration among public health agencies, communities and even academia to reduce tick populations and disease risk. Here, we describe a localized BDT infestation detected in 2024 in South Sacramento, California, an area with no prior detections and considerably farther north than locations of recently reported activity. We report on the multifaceted surveillance and control response launched by the Sacramento-Yolo Mosquito and Vector Control District (SYMVCD), in partnership with the Laboratory of Infectious Disease Ecology from the University of California, Davis (UC Davis).

All tick inspections, collections and residual treatments were conducted outdoors. All inspected residences measured approximately 60 × 170 ft (0.15 acres). During the initial inspection, tick surveillance was attempted using dry-ice baited traps. Each trap consisted of a 1 × 1-m white flannel sheet baited with approximately 500 g of dry ice placed inside an insulated 1-gallon can with a lid perforated by four holes to allow CO_2_ diffusion. The can was positioned at the center of the sheet, and traps were deployed for approximately 45 min before collection and inspection. No ticks were recovered using this method; therefore, subsequent surveys relied exclusively on environmental visual assessments. Residences were surveyed for ≥ 20 min if no ticks were found within the first 20 min, with a team of ≥ 3 people. At each property, inspectors systematically examined: (i) ground surfaces, (ii) cracks and crevices along exterior house walls, (iii) the perimeter fence lines and (iv) dogs present at the residence (when possible). Residents were also asked to check their own dogs for ticks. Ticks were collected using entomological forceps and placed into 70% ethanol for later identification and testing.

The SYMVCD received an initial complaint from Residence A located in South Sacramento, where the residents observed ticks both indoors and on two large dogs (Dogs A1 and A2), despite the use of oral tick prevention (Bravecto; Merck Animal Health, Rahway, NJ, USA). On 9 August 2024, SYMVCD personnel conducted an initial inspection at Residence A. No ticks were found in the front or backyard; however, along the shared fence line with Residence B, ticks were observed crawling toward Residence A, indicating Residence B as the likely source of infestation. On a closer inspection of Residence B, a medium-sized dog (Dog B) without consistent tick prevention was found to be heavily infested with ticks at all life stages. The property showed widespread infestation, with ticks present in the yard, on exterior walls, in the dog’s bed and on the ground. Both engorged and unengorged specimens were collected. In total, 160 ticks were collected and subsequently identified by entomologists from UC Davis and SYMVCD as *Rh. sanguineus* s.l.

Life stage and species determinations were performed under a dissecting stereomicroscope (model SZX16; Olympus Corp., Tokyo, Japan) using established morphological criteria [1, 25]. While we did not identify the ticks molecularly, a previous study confirmed the presence of *Rh. sanguineus* sensu stricto (temperate lineage) in California [26]. During this inspection, ticks were found in higher densities in grassy areas of the residence yard compared to the house walls and concrete patio floors. That same day, a residual treatment using Suspend® PolyZone® (0.03% deltamethrin concentration; Bayer Crop Science, Leverkusen, Germany), which contains 0.42 lb (190.51 g) per gallon (3.79 l) of deltamethrin, a type II synthetic pyrethroid-based pesticide, was applied to Residence A using a backpack sprayer (SOLO 4-gallon; Solo Inc., Newport News, VA, USA), and a follow-up treatment was scheduled for Residence B. The areas treated with Suspend® PolyZone® included outdoor ground surfaces, dirt and cement, cracks and crevices along exterior house walls, the perimeter fence lines and walkways, with the aim to cover as much of the outdoor areas of the property as possible.

**Fig. 1:**
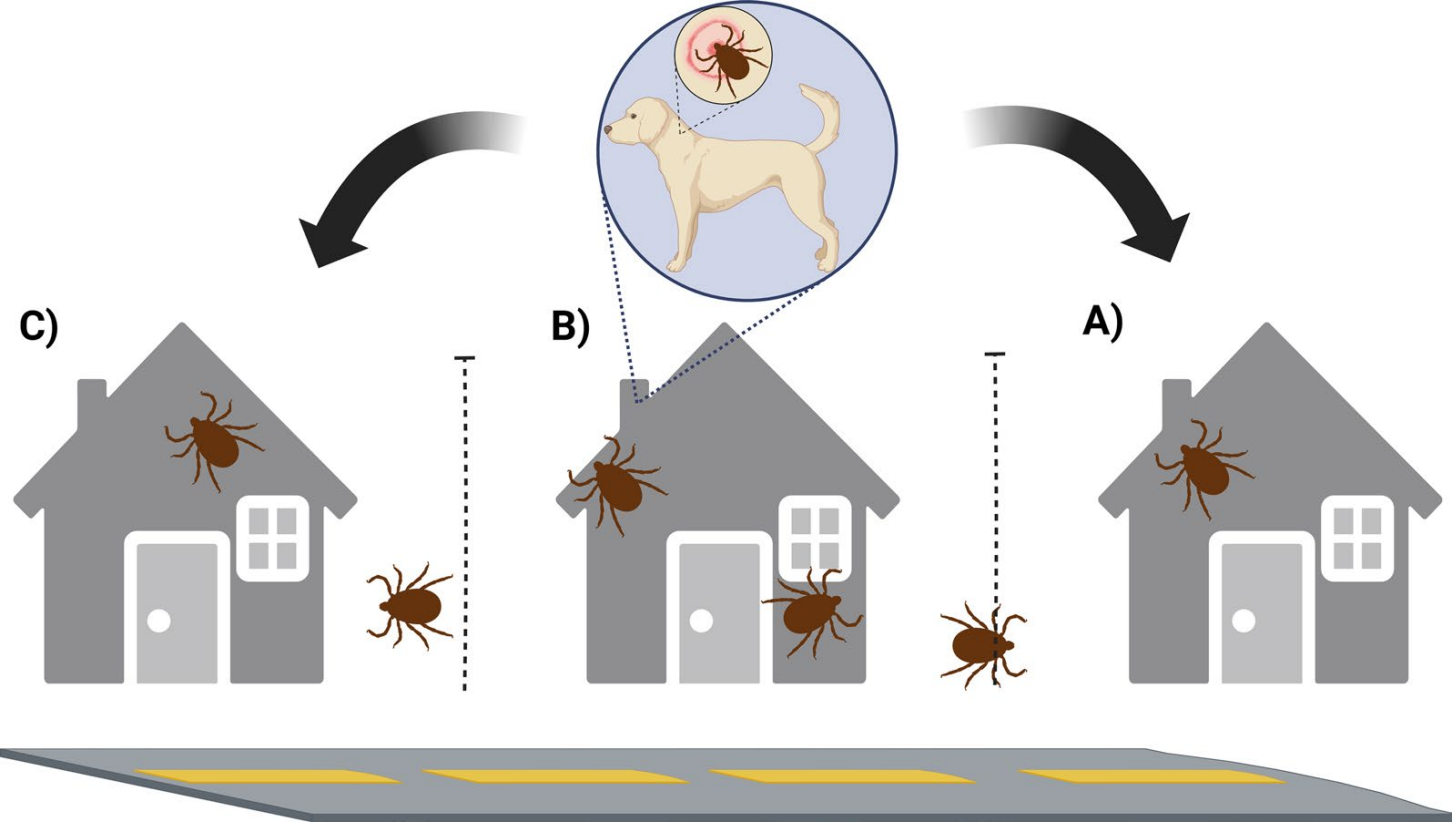
Schematic of the spatial arrangement of three adjoining residences that were affected by a localized *Rhipicephalus sanguineus* sensu lato infestation. Residence **A **(left) submitted the initial complaint; two dogs were present at the location. Residence **B** (center) was identified as the main source of infestation, where a heavily infested dog was identified. Residence **C** (right) showed milder infestation, with one dog present at the location. Dotted lines indicate shared fences, allowing likely tick migration. The properties were bordered by a road on the front yard and a ditch on the backyard side

On 14 August, SYMVCD canvassed 18 surrounding properties to assess the extent of the infestation. These included interviews with residents, visual inspections of pets and yards when permitted and residual treatments at the owners' request (Additional file 1: Supplementary questionnaire 1). We received responses from 12 residences and identified 14 dogs, five of which were already receiving tick and flea medication. We provided eight additional Seresto® collars (Elanco, Greenfield, IN, USA), containing imidacloprid and flumethrin, to dogs that were not previously treated, including Dog B. Additionally, Residence C, located on the opposite side of Residence B, also showed evidence of tick activity on a large dog (Dog C), based on owners reports to district personnel, although no ticks were collected during inspections, confirming that the infestation had spread to properties on both sides of Residence B (Fig. 1). No additional properties showed evidence of tick infestation. In total, three properties received residual Suspend® PolyZone® treatments. These included Residences B and C, as well as one additional adjacent property (treated at the owner’s request despite no ticks being found, not counted in Table 1). Residents were provided with information in Spanish and English on RMSF from the Centers for Disease Control and Prevention and best management practices for BDT control from the University of Florida Extension [27, 28].

**Table 1:** Summary of tick surveillance and control measures by residences identified as part of the infestation (2024–2025)

Status	Residence	Number of dogs	Action	Total number of ticks collected from the dogs and environment	Total number of residual treatments
Original complaint	A	2^a^	Suspend® PolyZone®	3	4
Main infestation	B	1	Suspend® PolyZone® and Seresto® collar provided	393	4
Secondary infestation	C	1	Suspend® PolyZone® and Seresto® collar provided	0	3

^a^Dogs were on tick prevention

Follow-up inspections continued on 16, 23 and 29 August, with the final inspection of the 2024 season completed on 12 September at all affected residences. Across the season, Residence B received two residual treatments (initial treatment plus one retreatment after continued detections), Residence A received two treatments (one preventive and one retreatment following owner reports), Residence C received one initial treatment but no retreatments since there were no further detections or reports, and the adjacent property received one treatment at the owner’s request. By the end of the 2024 season, SYMVCD had administered six residual treatments and distributed eight Seresto® collars according to dog body mass and whether the dogs were already on tick preventatives.

On 17 April 2025, the SYMVCD conducted its first inspection of the 2025 season. Fifty live adult BDT and no immature ticks were collected from Residence B, with the majority of the ticks collected from the environment, including along the shared fence line with Residence A and the exterior house wall cracks and crevices, likely indicating that BDT had survived the winter but that no new ticks had hatched. On 30 April, Residences A, B, and C were re-treated with an increased Suspend® PolyZone® application rate of 0.06% deltamethrin, and Dog B was fitted with a new Seresto® collar. During this visit, 30 live adult ticks were collected in the yard of Residence B. The ticks were found almost exclusively questing in the grass, and no immature or blood-fed ticks were collected. A subsequent inspection and treatment of properties A, B and C was carried out on 5 June using the higher deltamethrin rate, with only two unfed questing adult females collected. By 14 July, no ticks were observed at any residence.

Residences A and C were retreated as a preventive measure due to their proximity to Residence B, which continued to show tick activity. Homeowners at both residences reported seeing ticks on their properties, although no specimens were collected during district inspections. Treatments at higher rates were applied in early summer when tick activity is expected to increase with rising temperatures. These retreatments were intended to reduce the risk of re-establishment and to provide reassurance to the residents. No evidence of winter survival of *Rh. sanguineus* s.l. was documented at either location. A total of 136 ticks were tested by PCR [29, 30] at the UC Davis Laboratory of Infectious Disease Ecology and were negative for *R. rickettsii* presence. The total number of treatments, tick counts and actions taken are summarized in Table 1.

**Fig. 2:**
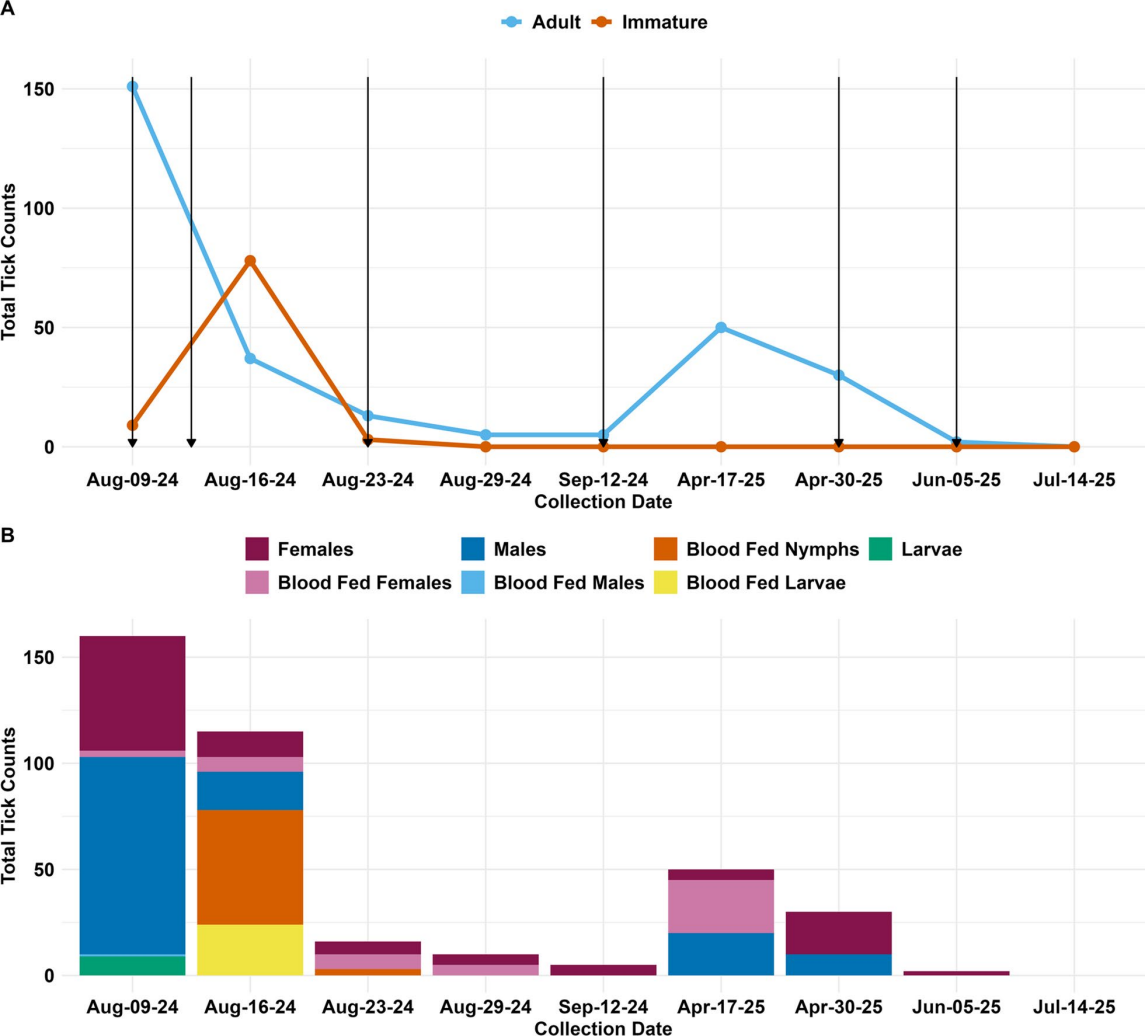
2024–2025 SYMVCD and UC Davis tick surveillance in South Sacramento. **A** Total cumulative tick counts for adults and immatures, regardless of sex, specific life stage (nymph, larvae) and blood-fed status across all residences. Arrows represent Suspend® PolyZone® (Bayer Crop Science) treatments regardless of the residence. **B** Total cumulative tick counts by life stage, sex and blood-feeding status. SYMVCD, Sacramento-Yolo Mosquito and Vector Control District

For the 2024 season, 1 week after the initial treatments, unfed adult male and unfed adult female tick populations decreased by 81% and 78%, respectively. Two weeks after the initial treatment, unfed males were completely eliminated and the unfed female populations had decreased by 89% relative to pre-treatment levels. Interestingly, immature ticks temporarily increased—by approximately eightfold following the initial treatment; however, after the second and third residual treatment, no immature stages were detected in any subsequent inspections (Fig. 2a). Two weeks after the third residual treatments, tick populations were reduced by 98% and during this time, the fourth and final residual treatment of the 2024 season was carried out. Despite the use of oral and topical acaricides on dogs, residual treatments in the environment and inspections, a tenfold increase in adult ticks was observed during the initial 2025 season inspection, 8 months after the initial intervention. However, following two additional treatments 1 month apart and using a higher deltamethrin rate, ticks were eradicated from all properties (Fig. 2b).

A multi-residence and multi-year infestation of BDT in South Sacramento highlights the challenges of controlling this important pathogen vector, particularly in urban environments. The absence of immature ticks in early spring indicates a potential interruption in the reproductive cycle; however, the presence of adult ticks implies survival over the winter period, likely associated with protected sites, such as wall voids, foundation cracks or animal shelters, and raises a possible concern for acaricide resistance. To address this, the SYMVCD implemented a monthly treatment schedule using a higher concentration of Suspend® PolyZone®, increasing the concentration of deltamethrin from 0.03% to 0.06%. While the label indicates that 0.03% is suitable for initial treatments, the 0.06% concentration is recommended for severe infestations requiring residual control [31]. Deltamethrin has been found to be effective against several tick species [32–34], including *Rh. sanguineus* s.l. larvae [35]. However, resistance in BDT populations to pyrethroids, phenylpyrazoles as well as ivermectin has been reported [36], and sublethal exposures may promote further resistance development.

Interestingly, after the initial residual treatment, Dog B remained untreated, suggesting that it was the main tick source as the number of ticks in the environment declined. However, following the third residual treatment, no immature stages were detected in subsequent inspections, including during the 2025 season. These results suggest that while residual treatments are effective against adult ticks, their impact on immature stages that are not yet host-seeking may be delayed. Newly hatched larvae and quiescent ticks often reside in protected microhabitats, such as soil, cracks and crevices, which may shield them from surface-applied insecticides. Additionally, ticks already attached to hosts at the time of treatment are less affected by environmental applications, allowing them to continue feeding either to molt into the next stage or, in the case of adult females, to reproduce and lay eggs. This underscores the importance of incorporating on-host treatments to prevent the next generation of ticks from developing. Because many ticks avoid exposure, either by remaining in quiescence, residing in protected microhabitats or hatching after treatment, repeated applications targeting both the environment and hosts are necessary to intercept them as they emerge and become active, ultimately disrupting the life-cycle and achieving sustained population control. Future efforts should look into potential resistance in local BDT populations, development of dose–response curves to optimize treatment strategies and persistence and the penetration level of residual treatment on different substrates.

Overall, the SYMVCD and UC Davis intervention combining multiple residual treatments with Suspend® PolyZone®, deployment of Seresto® tick collars on dogs and public outreach efforts resulted in substantial and effective tick control, ultimately leading to eradication of the infestation at the affected residences. Finally, this work also underscores the critical role of communication, community investment and education in the success of residential tick control programs. In many settings, the efficacy of interventions such as tick collars may be reduced due to loss, removal or lack of understanding of their importance. However, in this study, ensuring sustained access to replacement collars and providing bilingual education helped promote consistent use and community participation. These results emphasize that educational outreach and culturally relevant communication strategies are essential for maximizing the impact of tick control interventions.

## Supplementary Information

**Additional file1: Supplementary Questionnaire 1 detailing resident interviews conducted during the 14 August SYMVCD canvassing to assess the extent of the infestation.**

## Data Availability

Data are provided within the manuscript or supplementary information files.
