# Supplementary material for: Rhipicephalus sanguineus sensu lato infestation in an urban area in South Sacramento, California, USA
Source: Parasit Vectors. 2025 Oct 27;18:430. doi: 10.1186/s13071-025-07069-3 (PMC12560577; doi:10.1186/s13071-025-07069-3)
Supplement: Supplementary file 1 — Additional file1: Supplementary Questionnaire 1 detailing resident interviews conducted during the 14 August SYMVCD canvassing to assess the extent of the infestation. [file 13071_2025_7069_MOESM1_ESM.docx]

House address____________________________

Date____________

1- Do you have a dog(s)?

2- Have you found any ticks on your dog or in your backyard?

3- If so, how many and when did you see them?

4- Is your dog currently on tick prevention medication? What kind?

**5-Collect contact information from the resident and dog(s)**

**7. If ticks are present, ask if they would want treatment from the district.**

**8. If they have dogs offer a tick collar depending on size and weight of the dog, annotate below the collar provided (Large or small)**

**6-Distribute information on Brown dog tick and Rocky Mountain spotted fever**
